# Supplementary material for: Evaluating transformer-based models for structural characterization of orphan proteins
Source: Bioinform Adv. 2026 Aug 6;6(1):vbag215. doi: 10.1093/bioadv/vbag215 (PMC13563664; doi:10.1093/bioadv/vbag215)
Supplement: Supplementary file 1 [file vbag215_supplementary_data.pdf]

Supporting Information

Appendix A - p-value calculation of consensual secondary structure assignment

Given two different amounts of predicted alpha-helical, beta-sheet, or coil amino acids, we want to calculate how likely it is for the two different arrangements to agree on a certain number (or percentage) of residue-wise secondary structure annotations. We explicitly calculate all permutations corresponding to a fixed number of alpha-helix and beta-sheet (but not coil) consensual annotations (respectively n_H and n_B, given the total number of helix- and beta-annotated residues in both predictions, and the common length of the sequence (respectively H_1, H_2, B_1, B_2, and L).

Let us first solve the problem when only one variety is present. Given a sequence of length L and two predictions for the total number of alpha-helix residues $0 \leq H_1, H_2 \leq L$, how many permutations result exactly in n_H alpha-helix residues predicted by consensus? We have to account for all permutations for choosing n_H out of H_1 and H_2 residues, as well as the permutations between said elements. Moreover, we have to account for all permutations the remaining $H_1 - n_H$ and $H_2 - n_H$ alpha-helix residues will be paired to coil residues, their internal permutations, and the permutations of the remaining coil residues. This brings us to the following formula:

$$\begin{aligned}\#(n_H|H_1, H_2, L) = &\binom{H_1}{n_H}\binom{H_2}{n_H}\binom{L-H_2}{H_1-n_H}\binom{L-H_1}{H_2-n_H} \\ &\cdot n_H!(H_1-n_H)!(H_2-n_H)!(L-H_1-H_2+n_H)!\end{aligned} \tag{1}$$

When having two moieties H and B, the added difficulty is accounting for cross-assignments H-B (here n_{HB} if the residue is assigned H in the first and B in the second, and n_{BH} otherwise), which in turn depend on the assignment of B moieties. The resulting formula is

$$\begin{aligned}\#(n_H, n_B|H_1, H_2, B_1, B_2, L) = &\binom{H_1}{n_H}\binom{H_2}{n_H}\binom{B_1}{n_B}\binom{B_2}{n_B} n_H! n_B! \\ &\cdot \sum_{n_{HB}={}_{HB}}^{\mathscr{L}_{HB}} \sum_{n_{BH}={}_{BH}}^{\mathscr{L}_{BH}} n_{HB}! n_{BH}! (H_1 - n_H - n_{HB})! (H_2 - n_H - n_{BH})! \\ &\cdot \binom{H_1-n_H}{n_{HB}}\binom{H_2-n_H}{n_{BH}}\binom{B_2-n_B}{n_{HB}}\binom{B_1-n_B}{n_{BH}} \\ &\cdot \#(0|H_1 = B_1 - n_B - n_{BH}, H_2 = B_2 - n_B - n_{HB}, L = \mathscr{M})\end{aligned} \tag{2}$$

where

$$\begin{aligned} {}_{HB} &= \max(0, (H_1 - n_H) - (L - H_2 - B_2))) \\ \mathscr{L}_{HB} &= \min(H_1 - n_H, B_2 - n_B) \\ {}_{BH} &= \max(0, (H_2 - n_H) - (L - H_1 - B_1))) \\ \mathscr{L}_{BH} &= \min(H_2 - n_H, B_1 - n_B) \\ \mathscr{M} &= \max(0, L - (H_1 + H_2 - 2n_H) - n_H - n_B) \end{aligned}$$

and where the former single-moiety formula is used.

Supplementary figures

pLDDT correlation between ESMFold and OmegaFold

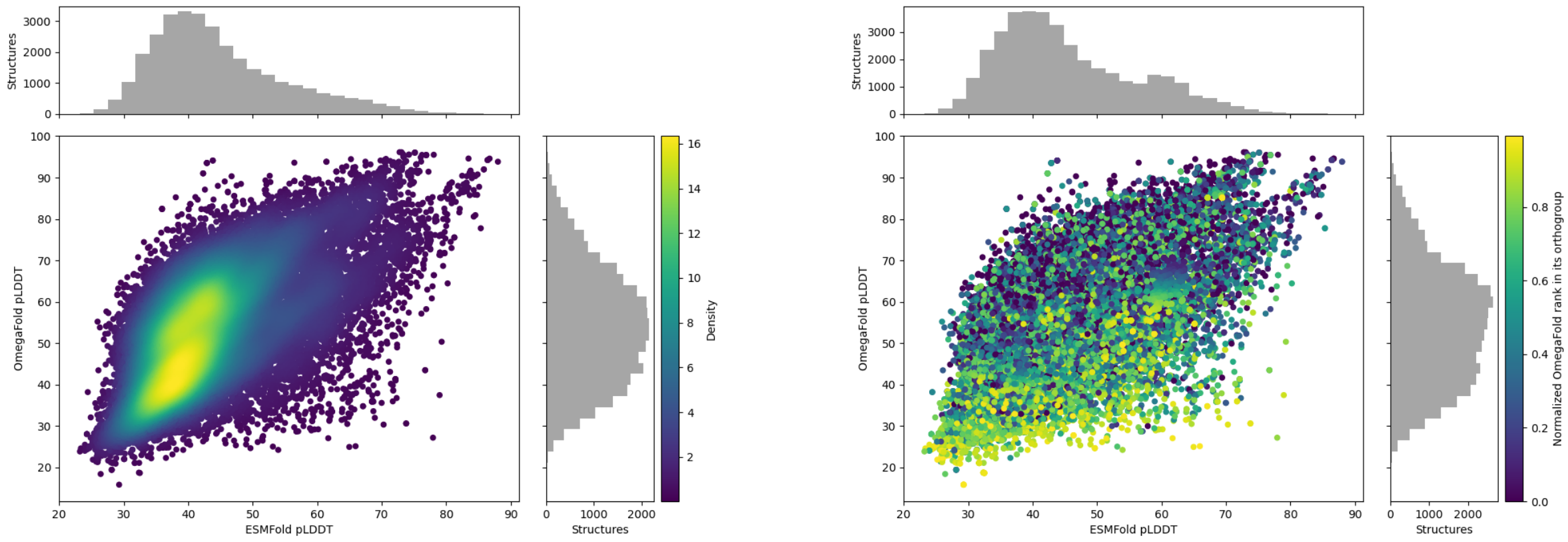

Figure S1: **pLDDT correlation between ESMFold and OmegaFold.** Right: Correlation between the orphan protein structure prediction pLDDT of ESMFold and OmegaFold. Left: structures are colored with respect to their score ranking within their orthogroup, according to OmegaFold.

Consensual secondary structure assignment

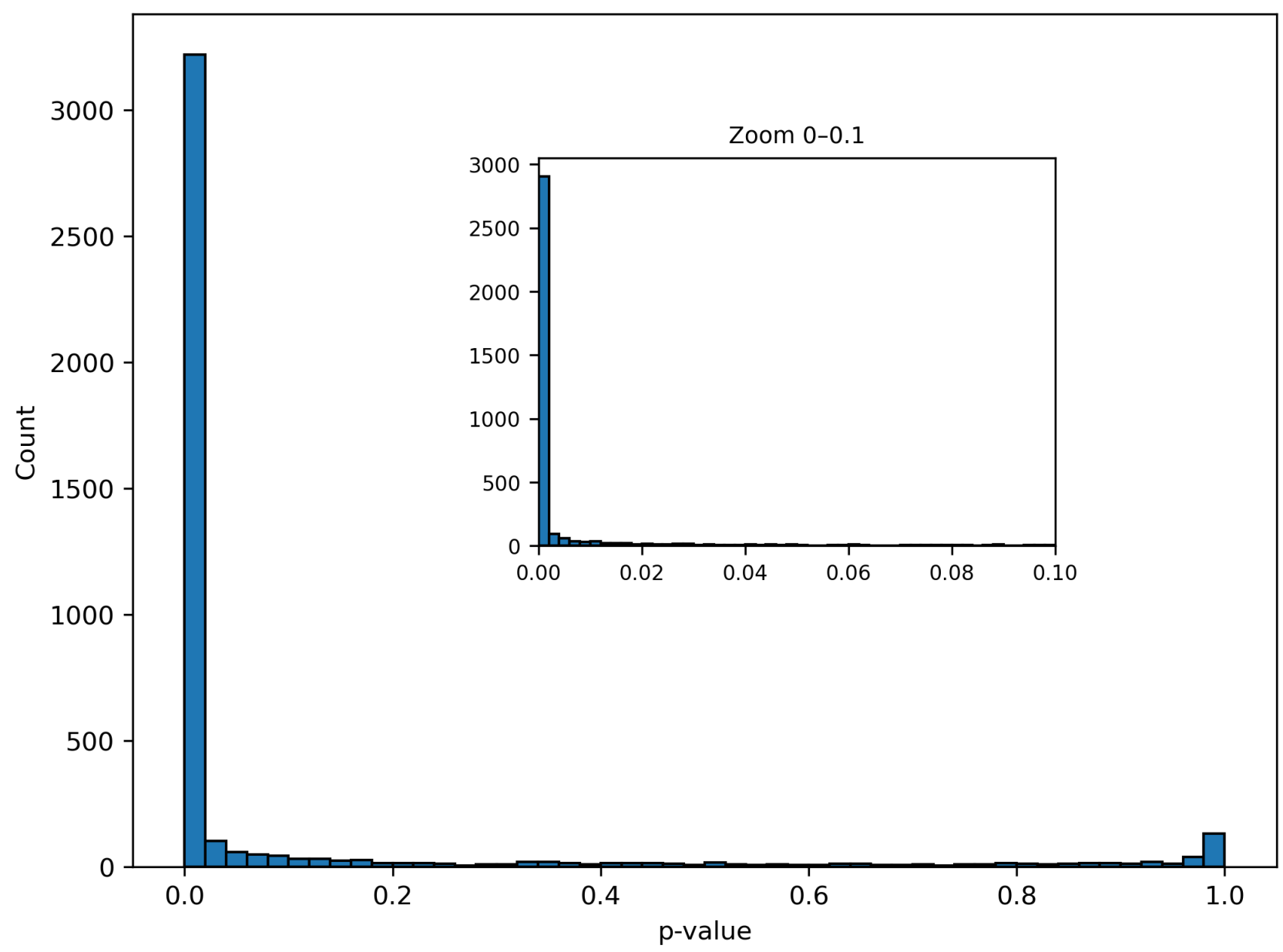

Figure S2: **Significance of consensual secondary structure predictions between AlphaFold2 and ESMFold**. The histogram reports the p-value of the consensus in the secondary structure prediction of each clade 1 orphan protein. The vast majority of cases falls below 0.001.

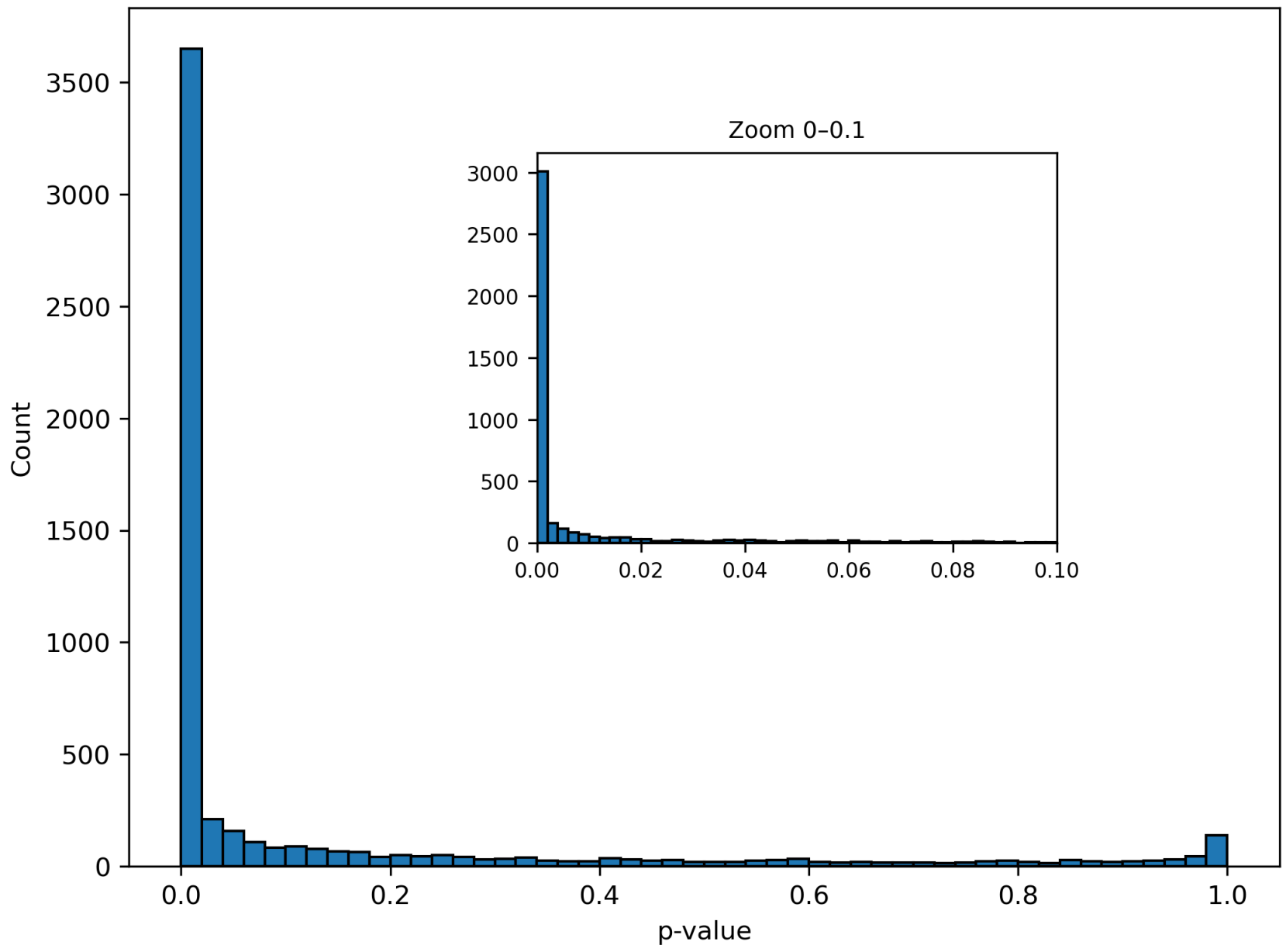

Figure S3: **Significance of consensual secondary structure predictions between ProtT5 and AlphaFold2**. The histogram reports the p-value of the consensus in the secondary structure prediction of each clade 1 orphan protein. The vast majority of cases falls below 0.001.

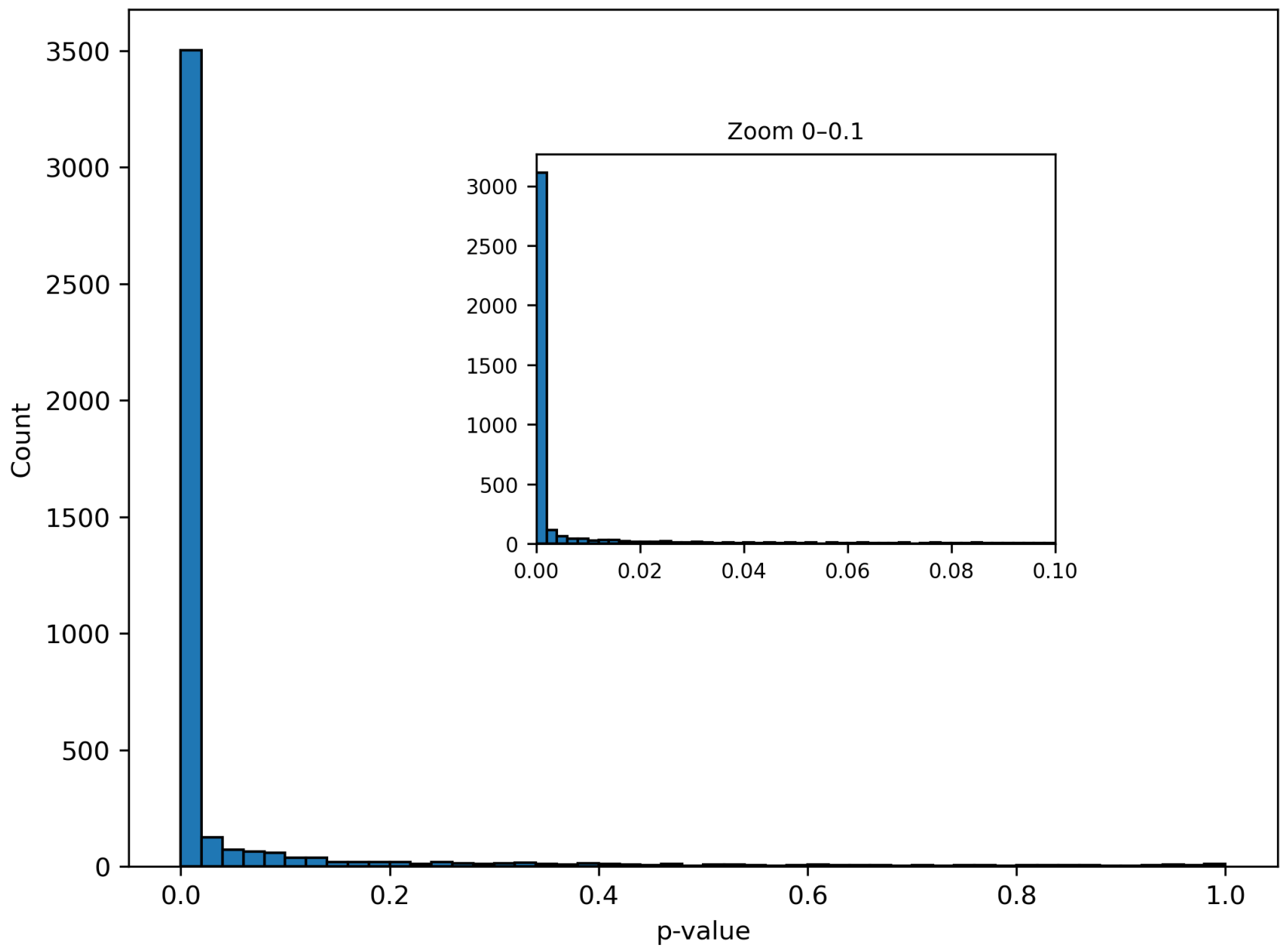

Figure S4: **Significance of consensual secondary structure predictions between ProtT5 and ESMFold**. The histogram reports the p-value of the consensus in the secondary structure prediction of each clade 1 orphan protein. The vast majority of cases falls below 0.001.

Secondary structure prediction

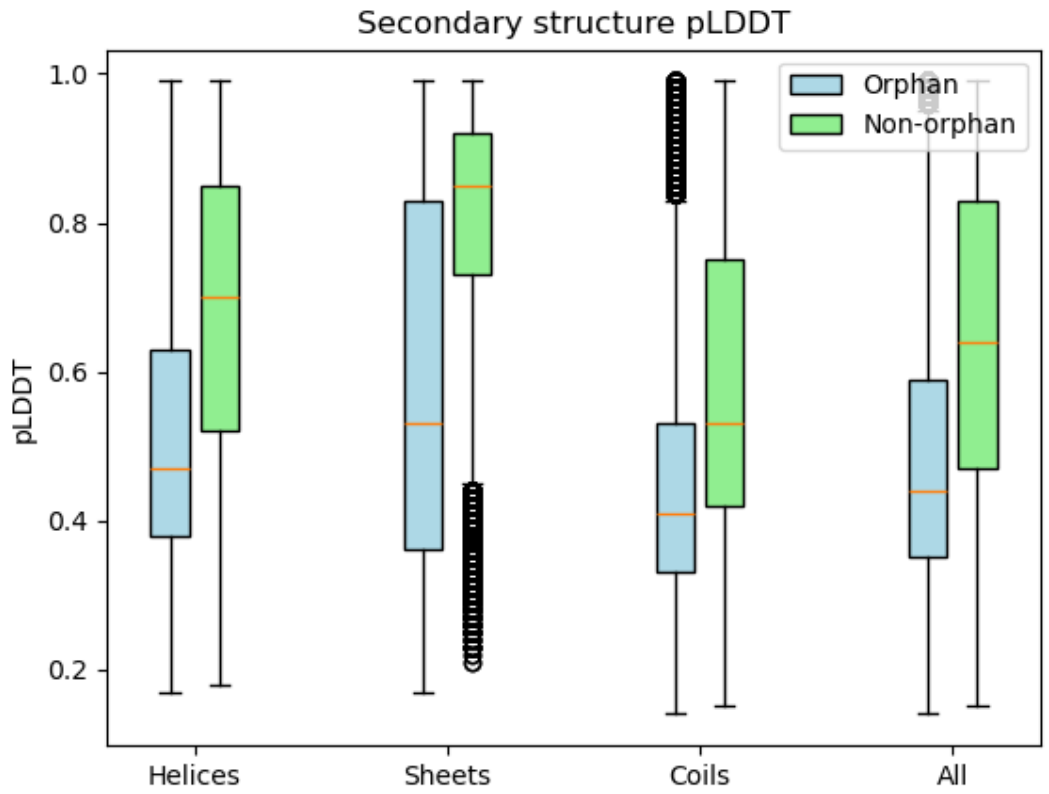

Figure S5: **pLDDT of secondary structure residues for ESMFold. The decrease in pLDDT is consistent throughout the presence or absence of secondary structure.**

	ESMFold	OmegaFold	ProtT5
AF2	75% 90%	76% 87%	65% 85%
ESMFold		83% 90%	72% 86%
OmegaFold			69% 84%

Table S1: **Fraction of secondary structure consensus prediction between any two predictors.** Orange: orphan protein dataset, blue: non-orphan protein dataset.

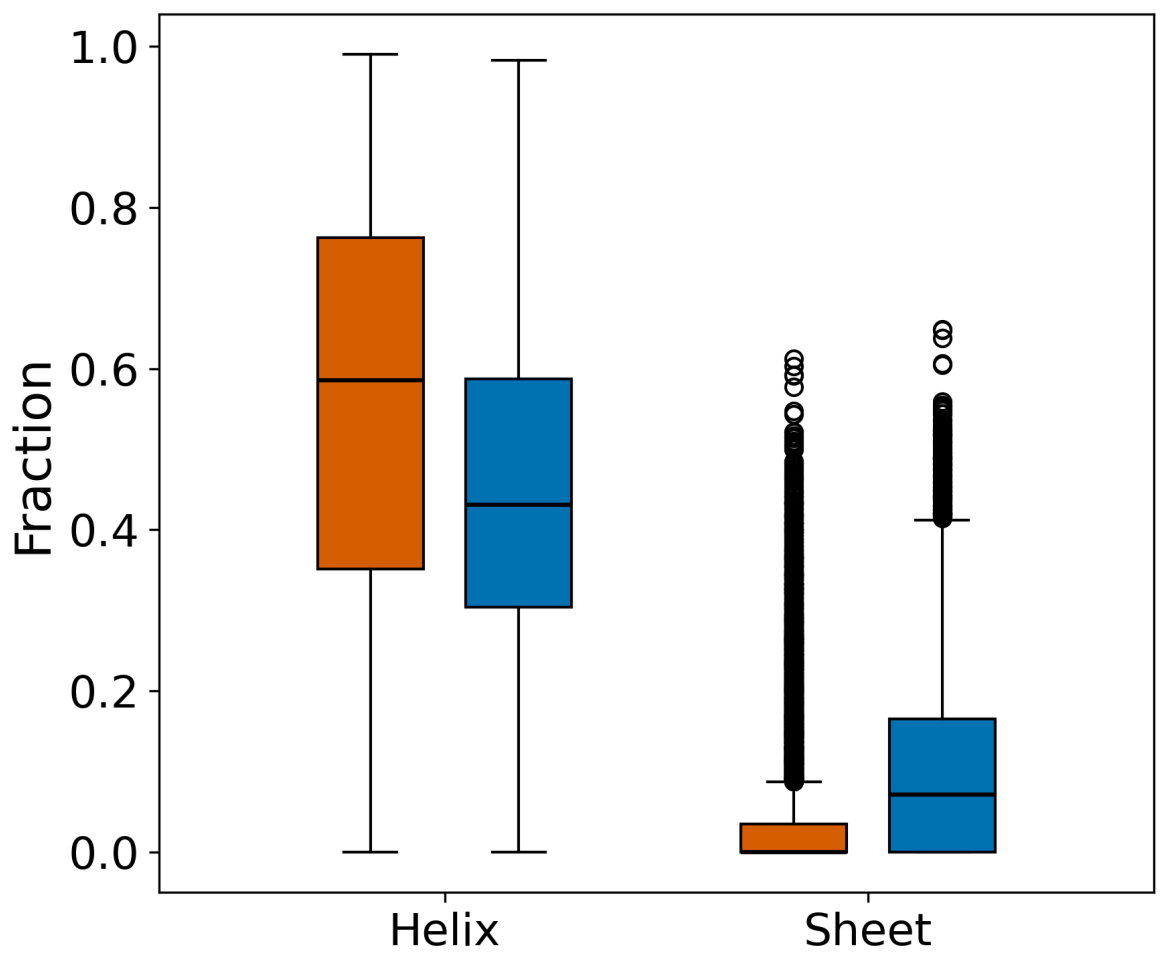

Figure S6: **Predicted secondary structure on orphans and non-orphans for OmegaFold**. Fraction of amino acids per sequence that are predicted as alpha helix and beta sheet, for orphan (orange) and non-orphan (blue) proteins.

Intra-orthogroup structure similarity

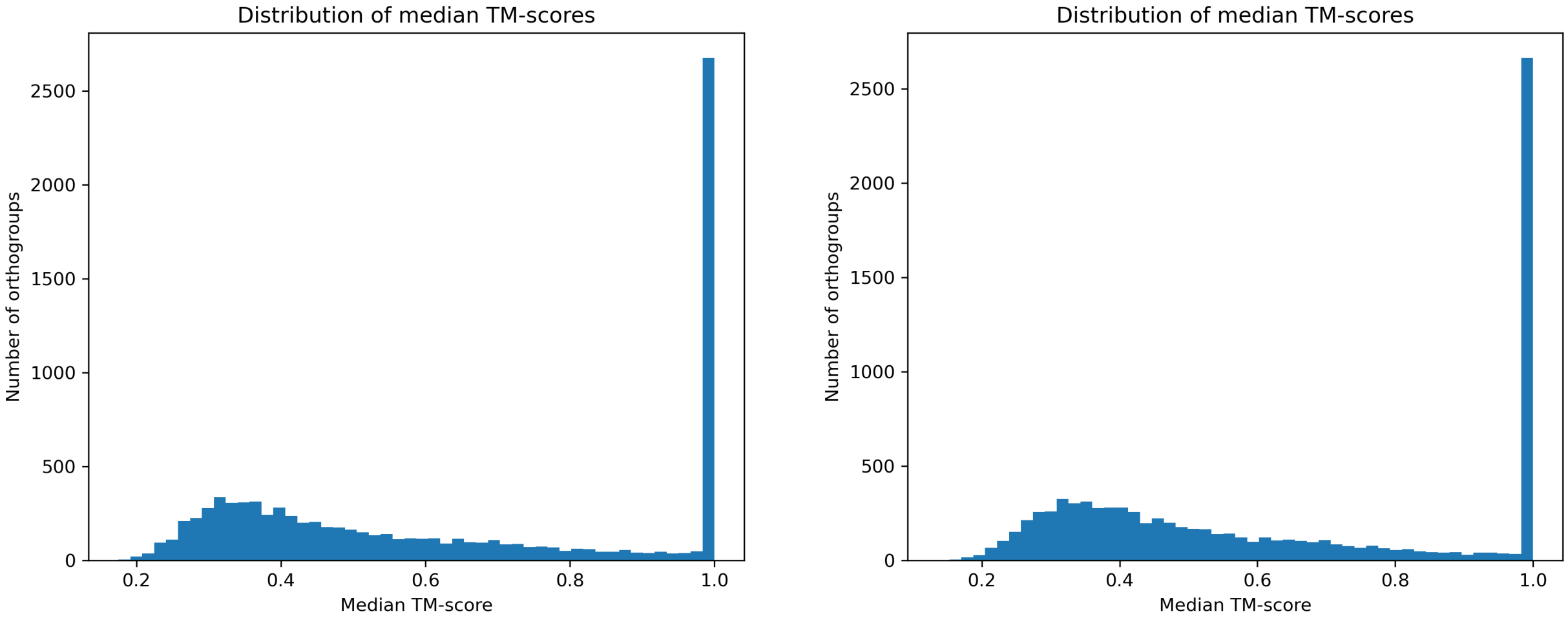

Figure S7: **Intra-OG structure similarity.** Median TM-score of intra-OG pairwise structure alignments within the same OG for ESMFold (left) and OmegaFold (right). More than 2,500 out 8,974 non-singular OG have identical predictions, whereas about 4,500 OG have TM-score < 0.5 (different fold).

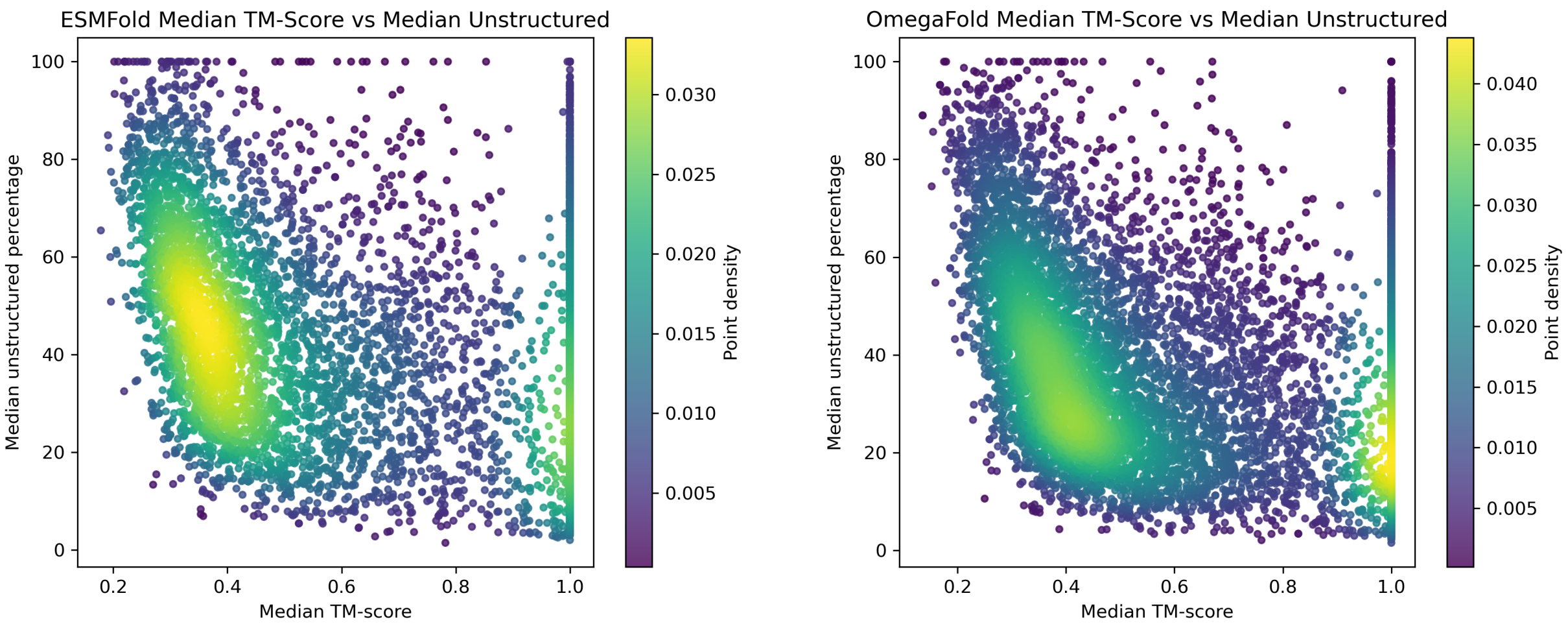

Figure S8: **Intra-OG structure similarity vs. disorder.** Median TM-score of intra-OG pairwise structure alignments vs. median percentage of non-structured residues (DSSP codes "B", "T", "S", "-") within the same OG for ESMFold (left) and OmegaFold (right). The median TM-score of the about 2,500 OG containing structures with different folds (median TM-score < 0.5) is highly anticorrelated with disorder, implying failure in the structure alignment.

Sequence length-restricted orphan protein dataset

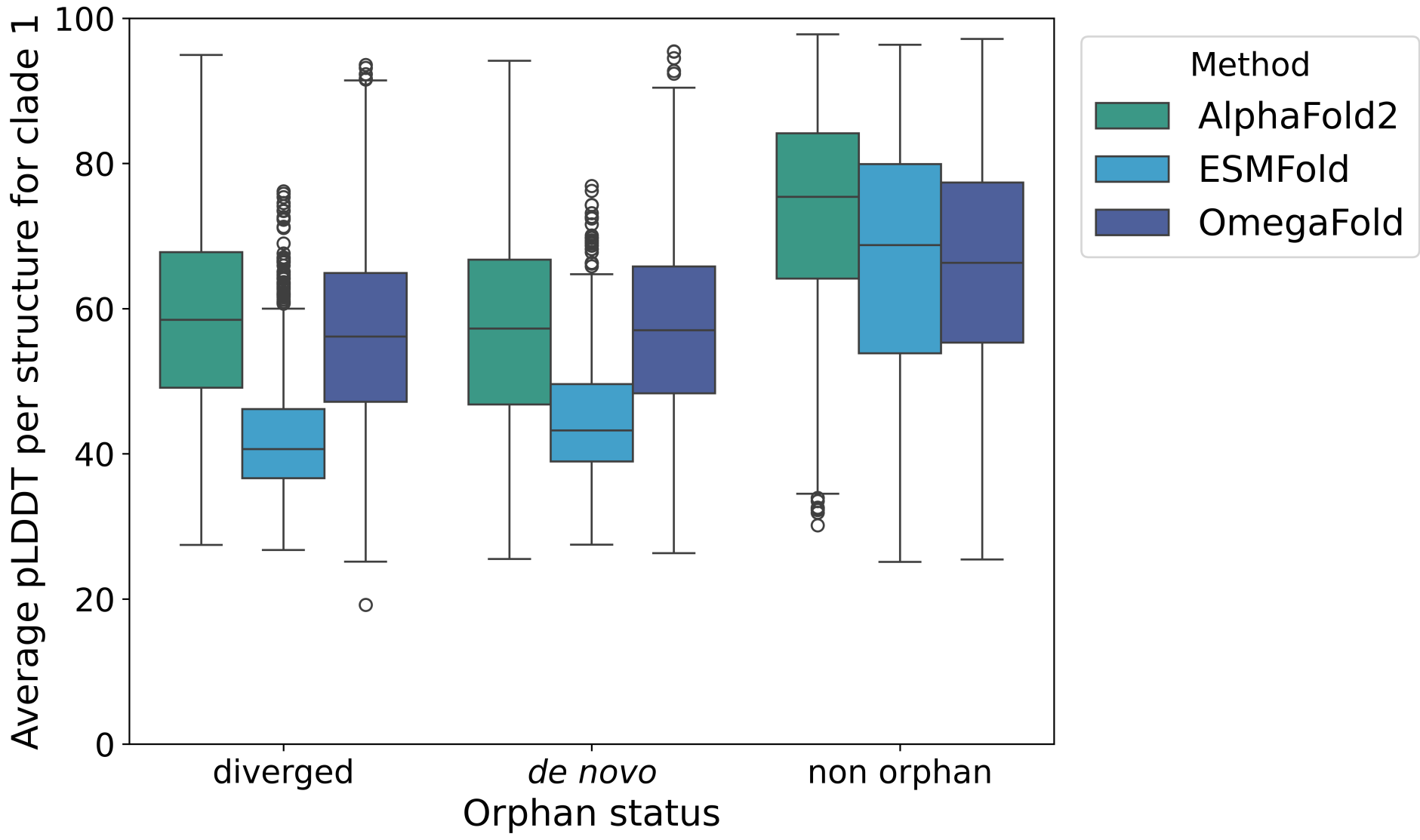

Figure S9: **Average pLDDT of orphan protein structure predictions for three TBMs on same-sequence-length datasets.** Average pLDDT of AlphaFold2, ESMFold and OmegaFold on confirmed diverged and *de novo* proteins. Average pLDDT of a selected same-size dataset of non-orphan proteins with matching sequence lengths. Sequence length is not a relevant factor for our observations.

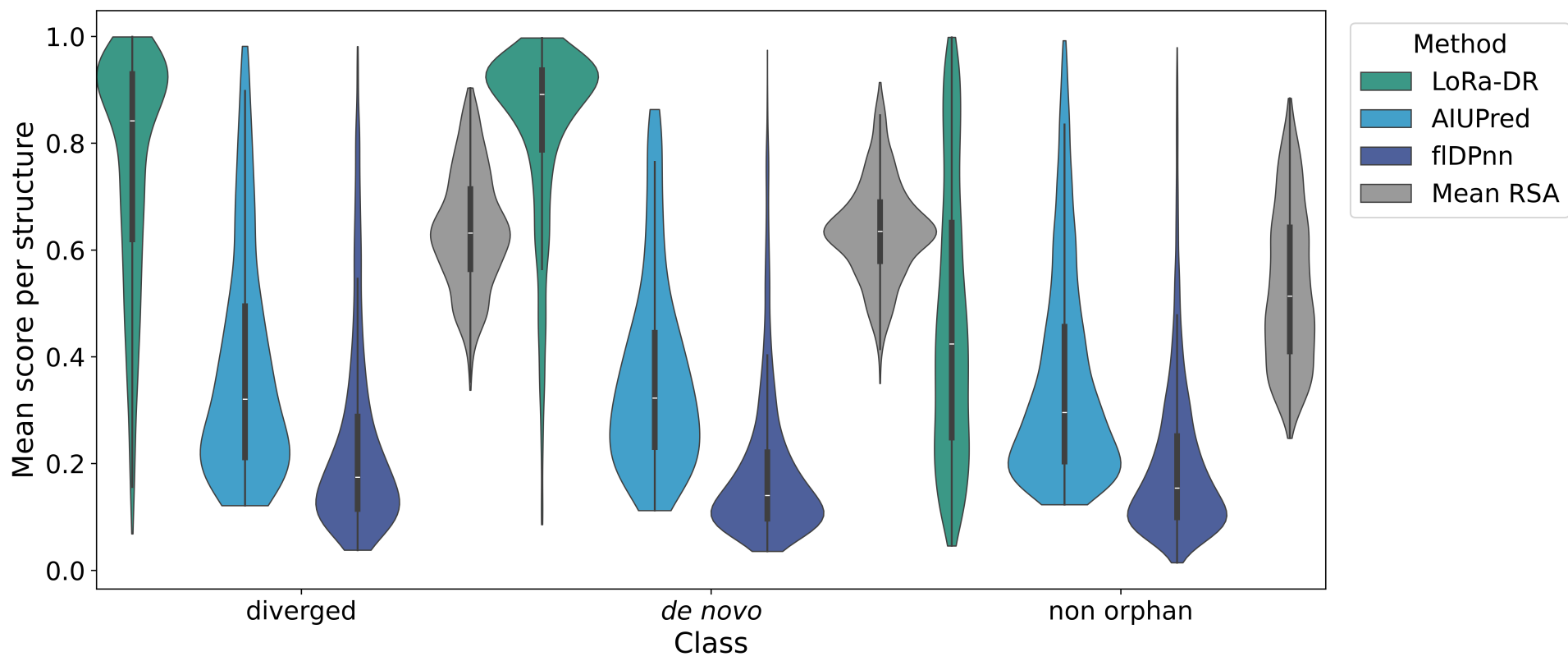

Figure S10: **Predicted intrinsic disorder for orphan and non-orphan proteins on same-sequence-length datasets.** Predicted intrinsic disorder with LoRA-DR, AIUPRed, flDPnn and AlphaFold2 (through RSA calculation). Sequence length does not play a significant role in this analysis.
